# Public engagement experiences in research using data administrative linkage to build a Social Disparities Index for Covid-19 in Brazil.

**DOI:** 10.23889/ijpds.v7i3.2089

**Published:** 2022-08-25

**Authors:** Maria Y. Ichihara, Adalton dos Anjos Fonseca, Mariana Rodrigues Sebastião, Denise Moraes Pimenta

**Affiliations:** 1 Centre for Data and Knowledge Integration for Health (CIDACS), Oswaldo Cruz Foundation

## Objectives

Public engagement is an important pilar to add new perspectives as take into consideration participants' interests in research. We aim to share the potential of Cidacs experience with data administrative linkage for creating the Social Disparities Index for Covid-19 (SDI-Covid-19).

## Approach

Dialogue is the core of our work. We invited policy makers, community groups and researchers based on their interest with the key themes of the project. Webinars, technical meetings, discussion groups, and individual conversations were the main methods explored to allow participants to know each other and to change experiences about social inequalities in Covid-19 pandemic. We used digital technologies to enable these interactions because of social distance measures. Website, posts on social media, guides, and minibiographies of participants were produced to share information periodically. Our engagement framework includes the following steps: inform, collect, consult, share, revise, disseminate, and evaluate.

## Results

We added new perspectives to SDI-Covid-19 suggested by the research participants during our events and individual conversations. Policy makers indicated a new level of information to make SDI-Covid-19 more useful for them in their daily work. We also discussed and interpreted some results with representatives of community groups from black movement, and slums. Besides that, they suggested some strategies to produce more accessible messages to support the scientific dissemination of research findings to civil society. Participants validated some information from SDI-Covid-19 and shared their impressions about how it can be used to design social policies. We connected 279 researchers, policy makers and representatives of community groups on two webinars; 29 policy makers on a technical meeting; 8 representatives of community groups on a discussion group.

## Conclusion

A more equitable and ethical research was possible with our engagement experience with SDI-Covid-19. The ideas added improved the quality of research, validated results, and supported dissemination. The use of linked administrative data has been recognized as useful resource for managers, community groups and researchers.

